# Indian rural women’s suicide beliefs and experiences: female suicidality as escape from and protest against a life usurped of agency

**DOI:** 10.1186/s12905-026-04676-9

**Published:** 2026-09-14

**Authors:** Debasruti Ghosh, Sunil K. Verma, Moneerah Mohammad ALmerab, Mohammed A. Mamun, Satchit Prasun Mandal, Saswati Bhattacharya, Saurabh Raj

**Affiliations:** 1 Department of Psychology, MDDM College, Babasaheb Bhimrao Ambedkar Bihar University, Muzaffarpur, Bihar 842002 India; 2 Department of Psychology, Vivekananda College, University of Delhi, Delhi, India; 3 Department of Psychology, College of Education and Human Development, Princess Nourah Bint Abdulrahman University, Riyadh, 11564 Saudi Arabia; 4 CHINTA Research Bangladesh, Dhaka, 1342 Bangladesh; 5 Department of Public Health, University of South Asia, Dhaka, 1348 Bangladesh; 6 Department of Psychology, Rajiv Gandhi University, Doimukh, Arunachal Pradesh 791112 India; 7 Department of Psychology, Christ University, Delhi NCR campus, Ghaziabad, Uttar Pradesh 201003 India; 8 Department of Psychology, Ramdayalu Singh College, Babasaheb Bhimrao Ambedkar Bihar University, Muzaffarpur, Bihar 842002 India

**Keywords:** Rural Women, Suicide, Narrative Inquiry, Cultural Scripts of Suicide, Women's Rights, Patriarchal Norms, Protest Suicidality

## Abstract

**Background:**

Suicide remains a significant public health concern in India, with women from rural areas bearing a disproportionate burden that remains under-researched. This study aimed to explore beliefs about suicide and understandings related to suicidality among women living in rural Bihar.

**Method:**

Using a qualitative narrative inquiry approach, four Focus Group Discussions were conducted across four villages in Bihar, India, where deaths by suicide among women had occurred during the previous two years. A total of 39 women aged 18–47 years participated who were purposively recruited from these communities. Six women (15.4%) reported a personal history of suicidal ideation. All participants had been exposed to suicide among women in their family or community, with 16 (41.0%) reporting exposure within their family and 23 (59.0%) reporting exposure within their village. Data were analysed using Riessman’s narrative framework.

**Findings:**

Participants described suicide among women as a response to persistent perceived violations of women’s human rights within family and community settings. Narratives highlighted restrictions on personal autonomy and decision-making, emotional and physical abuse, marital and reproductive pressures, economic dependence, and the stigma and exclusion faced by widows. Women were often expected to endure suffering in silence, with limited opportunities to seek support or challenge their circumstances. Within these contexts, participants frequently interpreted suicidality as both an escape from intolerable distress and a form of protest against conditions that denied women agency, dignity, and recognition.

**Conclusion:**

Suicidality among rural women in Bihar cannot be understood solely as an outcome of individual psychopathology. Rather, participants portrayed suicide as a culturally shaped response to enduring familial, social, and gender-based oppression. These findings suggest the need for community-based mental health support, gender-transformative interventions, and policy reforms that protect women’s rights, strengthen their social and economic resources, and expand opportunities for autonomy, safety, and well-being.

**Supplementary Information:**

The online version contains supplementary material available at https://doi.org/10.1186/s12905-026-04676-9.

## Introduction

Suicide remains one of the most pressing public health concerns of the 21st century, claiming over 700,000 lives globally each year [1]. Young women in India have higher rates of suicide than men, a pattern that contrasts with global trends across age groups, where women are generally less likely to die by suicide than men. As per the Global Burden of Disease Study, India alone accounted for 36.6% of the global deaths by suicide among women in 2016 [2], despite representing only a fraction of the world’s female population. In India, young women aged 15 to 29 are especially vulnerable, with suicide consistently ranked among the leading causes of death in this age group [2, 3].

Recent data from the National Crime Records Bureau further illustrates the pattern of suicide among women in India. In 2021, approximately 45,000 women died by suicide [4]. Among these, over half were identified as housewives, highlighting the importance of domestic, social, relational, and gendered contexts associated with this role important. Official statistics commonly classify the reasons for suicide among women under broad categories such as *family problems*, *marriage-related issues*, and *dowry disputes* [4]. However, such categories often conceal the underlying social conditions in which women experience discrimination, abuse, coercion, restrictions on autonomy, reproductive pressures, economic dependence, and other violations of their rights within family and community contexts. Moreover, framing these experiences as private family matters risks depoliticizing violations of women’s human rights and obscuring the broader structural and gendered inequalities that contribute to women’s suicidal vulnerability [5]. Research in low- and middle-income countries increasingly emphasizes the significance of context and culture as determinants of suicide among women [6]. In many cases, suicide reflects the cumulative impact of chronic gender-based inequalities and systemic violations of women’s human rights rather than merely individual or interpersonal difficulties. This perspective calls for moving beyond psychocentric explanations toward a contextual understanding of female suicidality that recognizes the role of structural disadvantage, patriarchal norms, and culturally embedded suicide scripts.

Undeniably, while mental health conditions such as depression and anxiety are important components of suicide risk, research in low- and middle-income countries increasingly emphasizes the significance of psychosocial stressors and cultural determinants. In many cases, suicide may be understood not only through individual psychopathology but also through accumulated suffering, systemic neglect, and emotional silencing [7–9]. Among women in rural India, these stressors are intensified by longstanding patriarchal norms, limited autonomy, and persistent economic and educational deprivation. Early marriage, restricted mobility, lack of decision-making power, and the prioritization of familial duty over individual well-being continue to shape the lives of many rural women, often leaving them isolated in their distress [10–12].

Cultural ideals such as *sahan-shakti* (endurance) and *samajhdari* (understanding) are frequently upheld as feminine virtues, equating silent suffering with moral strength. As a result, negative emotional expression remains unnoticed or suppressed, and women are discouraged from voicing pain or seeking help [13]. A cultural perspective on suicide, particularly suicide script theory, provides a useful framework for understanding these patterns. According to Canetto [14, 15], suicidal behavior is shaped by culturally available scripts that define who is more likely to become vulnerable to suicidal behaviors, under what circumstances, and with what meanings. These scripts are deeply gendered and reflect broader social norms, expectations, and power structures. In many contexts, women’s suicidal behavior is not merely an act of individual distress but a culturally scripted response to systemic constraints on women’s human rights, sustained by community and family norms. Within this cultural script, women's social, economic and psychological suffering is expected to remain unspoken, unacknowledged, and unsupported [15, 16]. While social norms encourage women to bear these pressures with composure, many are left without access to institutional support, emotional validation, or therapeutic resources [17].

Although feminist scholars have long highlighted the intersection between structural inequality and women’s mental health, much of the existing suicide literature in India remains focused on either clinical diagnosis or epidemiological data. Quantitative studies, while essential for identifying trends and at-risk populations, often fall short in capturing the emotional textures and lived realities of those most affected. Emerging qualitative research from low- and middle-income countries further supports this perspective. Studies from Sri Lanka and Nepal have shown that women’s suicidal behavior is often embedded in experiences of shame [18, 19], socially sanctioned oppression, and exposure to abuse [20]. Further, it may function as a form of communication, protest, or resistance within oppressive social structures. Suicidal behaviour is embedded in culturally shaped narratives of suffering, frustration, and anger, where its meaning and legitimacy are actively negotiated across class and gender positions. In a study from rural China, suicide among women functioned as a moral and symbolic rebellion and revenge against patriarchal family systems [21]. These studies highlight the importance of understanding suicide not only as an individual act but as a culturally mediated and socially situated phenomenon.

The present study seeks to address this gap by exploring how rural Indian women understand female suicide within their communities. Using a narrative inquiry approach, the study examines how women interpret the social, cultural, and gendered contexts in which female suicide occurs, with particular attention to restrictions on women’s autonomy, gender discrimination, abuse, and other violations of women’s rights within family and community settings. Rather than relying on individualistic or generalized explanations, this approach centres women’s narratives to understand the culturally shared meanings attached to female suicide and the ways in which social norms, structural inequalities, and culturally embedded expectations shape suicidal vulnerability. While some women may conform to social expectations of silence, duty, and endurance, others may experience a rupture in these expectations when familial, social, or economic pressures become overwhelming, leading some women to understand suicide as an escape [22]. Further, at times, it may be understood as a protest against a life marked by chronic invalidation and constrained agency [23]. By foregrounding women’s voices, this study seeks to move beyond psychiatric labels and illuminate the structural and symbolic contexts in which suffering is produced. Accordingly, the present study addresses the following research question: How do rural Indian women understand and make sense of suicide among women through their beliefs and experiences within their communities?

## Methodology

### Research design

This study used a qualitative narrative inquiry design, based on the epistemological assumption that human beings make sense of their world and construct meaning primarily through stories. Narrative inquiry was considered appropriate as the study aimed to understand how women’s life situations, challenges, and susceptibility to suicide are influenced by broader socioeconomic systems. Narrative inquiry acknowledges that stories are not merely representations of reality but are intricately woven into culture and influenced by memory, emotion, social context, and power dynamics. This method offered an advantage in centring the voices of Indian women and examining how they make sense of the social context of suicidality among women. It also enabled investigation of how violations of human rights may be related to rural women’s vulnerability to suicidal thoughts. 

The study adhered to the Consolidated Criteria for Reporting Qualitative Research (COREQ) guidelines to ensure transparency and rigor in reporting, and a completed COREQ checklist has been included as a supplementary file (Table S1).

### Research setting and participants

The research was conducted in four villages in Bihar, India, each of which had reported at least one death by suicide among women in the past two years. These settings were purposively selected due to their exposure to such incidents. Thus, the backdrop offered a contextually rich and emotionally charged context for exploring lived experiences of distress, hopelessness, and female suffering. Purposive sampling was employed to recruit participants. Initial access to the community was facilitated through the village *sarpanch* and *anganwadi* workers, who helped identify women residing in the selected villages and who were willing to engage in discussions on sensitive social and emotional issues. However, final inclusion was determined solely by the research team based on voluntary participation, informed consent, and relevance to the study objectives.

A total of 42 women were approached, of whom 39 consented to participate in the study. Inclusion criteria required participants to be adult women (18 years and above) residing in the selected villages and willing to share experiences or perspectives related to emotional distress, social pressures, and community understandings of suicide. This study aimed to capture the suicide beliefs and suicide experiences of community women. However personal history of suicidal behavior was not required for inclusion in the study as an informant. Information regarding participants’ exposure to suicidality, including self-reported ideation and exposure within close or community relationships, is summarized in Table 1.

**Table 1:** Participants exposure to suicide

Suicidal ideation/thought/exposure	Category	*N*
Suicidal Ideation	Yes	6
	No	33
Exposure to Suicide	Family member	16
	Village member	23

Four focus group discussions (FGDs) were conducted (one per village), with each group comprising 7 to 11 participants. The women participated in (FGDs) held between 21 February, 2024 and 4 November, 2024. While the age range of participants (18–47 years) was relatively broad, efforts were made to ensure contextual and relational homogeneity, such as shared village setting, similar socio-cultural backgrounds, and pre-existing familiarity among participants. This approach was considered essential in fostering a safe and open environment for discussing sensitive issues. This research setting allowed for both diversity of voices and group cohesion during discussions. The participants included 21 unmarried participants, 16 married women, and 2 widows, with ages ranging from 18 to 47 years. Informed consent was taken from the participants and they were also instructed that they had the right to withdraw at any point of the discussion.

### Data collection

Data collection was carried out by a female researcher (DG) familiar with the cultural and linguistic background of the villages, which helped in building trust and rapport. All FGDs were conducted in the local dialect, audio-recorded (with consent), and later transcribed and translated into English for analysis. Non-verbal cues, gestures, and emotional reactions were documented through field notes.

### Focus group discussion guide

While narrative inquiry often relies on in-depth interviews, this study employed FGDs to capture the collective and socially constructed nature of women’s narratives in rural contexts. In such settings, experiences of distress and suicidal thoughts are embedded within shared cultural norms and relational dynamics. FGDs enabled participants to co-construct meaning through interaction and shared reflections. This approach also facilitated the exploration of interactional and performative aspects of narratives, while fostering a sense of solidarity and psychological safety for discussing sensitive issues.

A semi-structured FGD guide was developed using open-ended, culturally sensitive, and emotionally safe questions to facilitate storytelling rather than direct interrogation. The questions were organized into key thematic areas: daily life (e.g., “Can you share what a typical day in your life looks like?”), emotional distress (e.g.,“What kinds of problems or pressures do women in your community usually face?”), and gendered expectations (e.g., “What is expected from women in terms of behaviour, responsibility, and tolerance?”), expression of distress, silence and support, beliefs about suicide, community responses, meanings of suicide, and coping alternatives. A particularly sensitive section focused on beliefs about suicide and community responses to suicide, explored through carefully worded questions such as, “In your opinion, what factors may contribute to suicide among women?” and “How do people in the community react when such incidents occur?” Each question aimed to elicit narrative, reflective responses in a non-judgmental and respectful environment. (Supplementary Table S2).

### Ethical considerations

Every participant gave their verbal (wherever literacy was an issue) or written informed consent. The Ramdayalu Singh College research committee gave its approval for the study (RDSPSY/21-2024). Pseudonyms were used to maintain confidentiality and anonymity. Given the sensitive nature of suicide-related research in low-resource settings, particular attention was paid to ethical and contextual challenges, including managing participant distress, ensuring cultural sensitivity, and minimizing potential harm. As highlighted by Mugisha et al. [24] conducting qualitative research on suicide in a low-income country requires careful navigation of stigma, emotional vulnerability, and limited access to mental health support systems. These considerations informed the study’s approach to data collection, participant safety, and referral practices. Additionally, participants were made aware of their freedom to leave at any time.

### Data analysis

In order to guide the analysis, the study drew on Riessman’s (2008) model of narrative analysis [25], which attends to four key dimensions:

- *Structure* – This determines the organization of the narratives that includes, its temporal sequencing, coherence, and narrative arc.
- *Content* – This consists of the verbatim, the themes and meanings expressed within the narrative.
- *Performance* – Thus is how the narrator presents the story, that is determined by the tonality, affect, emphasis, and interaction with the listener.
- *Context* – This explains the factors especially, the broader social, cultural, and relational settings in which the story is told and received.

Riessman’s framework gave an opportunity to evaluate each narrative in a nuanced, and multilayered manner, considering not only what women disclosed but also how and why they told their stories in particular ways. This method was useful in eliciting insights on social norms, the female dynamics of emotional expression, and the symbolic significance of speech and silence in rural areas. The analysis focused on participants’ narratives reflecting community-level beliefs, experiences, and meanings of suicide, rather than accounts limited to personal suicidality or direct bereavement. Additionally, it also acknowledges the interpretive nature of human experience, where individuals draw upon cultural meanings, social norms, and shared belief systems to make sense of their lives. It highlights women’s understanding of suicide are actions of resistance, survival, and self-definition rather than merely descriptions of sickness.

This study made use of the narrative analytical framework, which emphasizes thematic, structural, interactional, and contextual dimensions of storytelling. Using Labov’s (1972) narrative components as a guide, structural analysis looked at how participants organized and interpreted their experiences [26]. Women’s co-construction of meaning in group situations was observed in order to analyse interactional and performative features. These observations were made through a note of various gestures such as nods, sighs, interruptions, and shared cultural metaphors as recorded in the field notes. Contextual analysis explored underlying gender norms, silence around emotional pain, and stigmas linked to widowhood and beliefs about suicide. The analysis also captured emerging patterns and divergences, revealing both shared suffering and moments of resistance, where women challenged or redefined cultural expectations. The themes generated, coding and findings have been presented in Table 2.

**Table 2:** Summary table for themes and nature of narratives

Main Theme	Beliefs about What Triggers Female Suicide	Emotional Tone	Constraining Femininity Scripts	Outcome / Consequence
Theme 1: Normalization of Women’s Suffering within Family and Community Life	Emotional pain is seen as ordinary and not requiring attention	Resignation, despair	Crying is natural for women; suffering is expected and inherited	Emotional invisibilization; internalized helplessness
Theme 2: Enduring Chronic Distress in Silence under Expectations	Suppressed distress builds up silently until it becomes unbearable	Sympathy, helplessness	Infertility as shame; silence is strength; tolerance is womanly virtue	Suicidal ideation; emotional collapse
Theme 3: Restrictions on Women’s Autonomy and Life Choices	Denial of decision-making power in education, marriage, and roles	Regret, psychological exhaustion	Early marriage; obedience over ambition; caregiving as female duty	Loss of self-determination; suppressed aspirations; suicidal thoughts
Theme 4: Intergenerational Reproduction of Patriarchal Gender Norms	Patriarchal values passed on through maternal influence	Cognitive dissonance, irritation	Boys earn, girls serve; respecting men ensures stability; tradition is unquestioned	Reproduction of gender inequality; muted resistance
Theme 5: Male Usurping Females of their Opportunity for Education and Work and Coercing Women into Obedience	Capable girls denied mobility and freedom under the guise of protection	Anger, frustration, misery	Girls should stay home; domestic readiness over intellectual growth	Educational compromise; emotional suppression
Theme 6: Social Isolation and Lack of Support in Times of Crisis	Emotional suffering is invalidated by family; no avenues for support	Grief, loneliness, hopelessness	“Learn to adjust”; widows blamed and isolated; abuse minimized as normal	Suicidal ideation or completion; deepened psychological vulnerability

### Researcher positionality

A female researcher who knew the local language served as the primary data collector, with help from an associate who was proficient in the local tongue and familiar with the customs of the area. Participants were encouraged to relate intensely personal and emotionally charged experiences without fear of judgment because she was a woman and had culturally sensitive presence, which created a secure and trusting setting. The researcher adopted an empathetic and non-intrusive approach, which was crucial in facilitating open dialogue on sensitive issues such as emotional distress, societal pressure, and suicidal thoughts. Throughout the study, she maintained reflexive awareness of power dynamics, positionality, and ethical responsibilities in order to warrant respectful and responsible engagement. In order to record positionality, emotional reactions, and changing interpretations throughout the data collecting and analysis process, the researcher kept a reflexive journal.

Throughout the study process, a number of tactics were used to enhance the study’s rigor, dependability, and credibility. To improve the breadth and coherence of findings, triangulation was employed by cross-checking insights from several sources, such as FGD transcripts, thorough field notes, and debriefing meetings with fellow researchers.

## Results

### Theme 1: Normalization of women’s suffering within family and community life

The social normalization of women’s emotions, especially grief, sobbing, and despair, to the point where their underlying causes are frequently disregarded or dismissed, is encapsulated in this theme. A woman crying may not be shocking or frightening in many traditional contexts; rather, it is viewed as a normal, almost unremarkable behavior that is an extension of her female functionality. As a result, the underlying psychological distress, existential anguish, or emotional roller coaster that may give rise to such manifestations is often ignored or disregarded.

Several participants expressed similar views. One participant, a 21-year-old unmarried woman,

“*Even if we cry*,* we wail*,* others think women weeping is okay*,* what goes inside hardly matters… (sighs) Who will understand that we are hurt? For men*,* if women are crying*,* then it is normal. For other women*,* women crying is explained always in a way ….like our lives are like this only from centuries*,* it will remain like this.” (Others also sighed.)*

This sentiment was widely echoed within the group. The participant’s words here are expressive of a double layered neglect i.e., the external trivialization of women’s pain by society, and the internalized belief among women themselves that suffering is their fate. The normalization of women’s emotional expression reinforces a debilitating social narrative, that their distress is not extraordinary, and therefore does not require acknowledgment or intervention. This results in the “*emotional invisibilization” *(Table 2) i.e., a state that reflects an unawareness of their inner world which is characterised by the fear, loneliness, and despair that lie beneath visible emotions.

The participant’s reflection on other women’s perspective that “*our lives are like this only from centuries*” highlights an intergenerational pattern of helplessness or a resignation state where emotional suffering is passed down not only as experience but as expectation. Emotional pain becomes woven into the fabric of female identity, reinforced through both social response and self-perception.

This theme also challenges the ways that women’s emotions have historically been both magnified and restricted by typical male-female roles. Cultural prejudices frequently portray women as more “emotional,” however this term is used to minimize rather than to comprehend their feelings. It also reflects that instead of being placed within broader socio-cultural, relational, or structural difficulties, their suffering is pathologized or patronized.

Essentially, the normalization of women’s emotional pain has the silencing effect of making their suffering seem unimportant enough to need immediate attention. It perpetuates a culture where women still bear psychological and emotional problems in silence, with few chances for empathy, healing, or validation.

### Theme 2: Enduring chronic distress in silence under expectations

This theme reflects a shared understanding among participants about the perilous buildup of unaddressed emotional distress that often goes unnoticed in the lives of women, especially within patriarchal and oppressive familial systems. In many situations this pain in silence is internalized due to fear, shame, or cultural conditioning to an extent wherein, the emotional burden becomes overwhelming. This silent accumulation of grief, anger, humiliation, and helplessness gradually fills an invisible vessel within, pushing them toward a breaking point.

As Participant 4 (married, age 27), metaphorically expresses, while describing her friend:

“*Women keep their views lot inside; they have immense capability. They never know when that pot inside becomes full and starts to overflow. It begins to affect their thoughts*,* their behavior…now if it overflows there is no other way out other than ending the suffering by dying. She used to listen a lot*,* for not having baby….people said so many things to her….her in-laws taunted her for not being able to carry their generation further…they also instigated her husband to separate from her and marry someone else…they used to have frequent fights…her family insisted to stay in the relationship as it might work-out. but in vain*,* it got worse day by day.she might have thought dying is easier than seeing all this…”.*

The metaphor of an “overflowing pot” poignantly illustrates a woman’s internal struggle as she silently bears infertility, emotional neglect, humiliation from in-laws, and her husband’s insensitivity. Trained to remain silent in a society that devalues women’s suffering, she suppresses her pain until it accumulates beyond containment. The overflowing pot marks the breakdown of emotional resilience, bringing intense feelings of loneliness, despair, and helplessness. In such moments, self-harm or suicide may appear as the only escape from a life of silent suffering.

Additionally, this theme highlights how women’s mental health problems are less prioritized. Their suffering is generally ignored until a crisis arises, by which time it is usually too late to intervene. The absence of safe emotional outlets and the normalizing of suffering without protest create a psychological environment in which the only ways to feel better about pain are to die or remain silent. Further, the participant’s story employs metaphor in a compelling manner. It suggests that while women may not openly or clinically express their grief, they often do so by employing culturally embedded metaphors and symbols while silently waiting for someone to listen and understand. Sadly, people in their immediate vicinity usually overlook or fail to recognize these signs.

This theme essentially emphasizes how emotional suffering builds up over time and the terrible results of neglecting its slow accumulation. These signs of helplessness and rejection act as triggers of suicidal behavior. Before the “overflow” becomes lethal, it urges more awareness of the subtle indicators of suffering and a societal change that promotes candid emotional communication, mental health assistance, and systemic compassion.

### Theme 3: Restrictions on women’s autonomy and life choices

This theme explores the profound emotional and psychological consequences that arise when women lack independence of decision-making process in their own lives related to, marriage, motherhood, and caregiving roles. Loss of autonomy is not merely a restriction of physical freedom, but a deeply internalized sense of helplessness, where one’s choices, dreams, and desires are systematically overridden by familial or societal expectations. In such contexts, women become passive actors in the narrative of their own lives.

Participant 36 (married, age 20) offers a testimony of this experience:

*“I myself feel helpless sometimes, I was third in order of my siblings and after me there are two sisters and a brother. My father got me married at the age of 16, I scored 81% in 10th, I was told that I could study further (tear in eyes). It has been 4 years now, and I have a kid and two grandparents-in-law and parents-in-law, who will take care of them? My husband hardly understands all this. My dream of studying has taken a backseat, I feel overburdened. At times, it feels too much….I wish I could end it.”*

This narrative is steeped in regret, frustration, and oppressive emotional burden. The participant’s life path was determined by patriarchal choices i.e., early marriage, motherhood, and multigenerational caregiving. Thus, reflective of ignorance of their academic potential and ambitions for further study and strictly sticking to traditional roles. This left little opportunity for personal development or goal-pursuit. The emotional damage is exacerbated by the subtle betrayal of being assured she could study but then being denied that opportunity. Her unmet dreams end up serving as a persistent reminder of the life she could have led.

The absence of spousal understanding and the emotional neglect within the family unit further compound her distress. Her identity is reduced to that of a wife, mother, and daughter-in-law that hardly left space for selfhood. The loss of educational opportunity is not just the loss of a degree rather, it symbolizes the erasure of agency, ambition, and future possibilities.

This theme also underscores how the structural and cultural expectations placed on women often lead to emotional entrapment. The overwhelming responsibilities and lack of support systems create psychological exhaustion, sometimes pushing women to contemplate death as a form of liberation from a life they never chose. There were moments of collective silence when women described how they are often denied the right to dream, to decide, and to develop, the internal erosion of self becomes inevitable.

*As described by Participant 18 (unmarried) “ Girls and boys cannot be same, everything is so much societally governed…. what is to be done by girls and what not… how much to talk, at what pitch to talk, how to walk…when to go out, when not to go….boys don’t have to follow much role…independence is so different for both…every time someone is observing you….people have so much time to see you growing up…and most of it before a girl enters her 10th class, people start asking for marriage.one of my friend she was in her class 12th and her parents were eager to get her married of…she was so helpless, so much in pressure that she was thinking to drink the rat poison….For two months she was in tension…her aunt made her parents understand that they should wait till she finishes her 12th.*

The narrative speaks to a wider sociocultural issue where girls’ potential is celebrated only up to the point where it does not challenge traditional norms. Promises of education and self-betterment are dangled as temporary affirmations, but easily withdrawn under the weight of patriarchal control. This creates a deep inner conflict between what they hoped for and what they are forced to settle with.

This account illustrates how lack of autonomy can push young girls into extreme psychological distress. The threat of being married off before completing basic education triggers suicidal thoughts, reflecting the magnitude of pressure and helplessness felt when one’s life decisions are dictated by others. While her friend was temporarily saved by an aunt’s intervention, the narrative reveals how close many girls come to emotional collapse due to the weight of imposed expectations.

Cumulatively, these narratives illustrate that loss of autonomy is not a single moment of control, rather, it is a continuum of experiences where dreams are deferred, voices silenced, and lives shaped by others. This theme calls attention to the urgent need for systemic change in how autonomy is viewed and granted to girls and women that is not as a luxury or privilege, but as a fundamental right.

### Theme 4: Intergenerational reproduction of patriarchal gender norms

This theme delves into the deeply rooted process through which gender norms, expectations, and roles are passed down from one generation to the next, primarily through family socialization, especially maternal influence. Women across the groups shared that often they are both the recipients and transmitters of patriarchal values, socializing their daughters into the same roles and ideologies that once limited them. This cycle of conditioning reinforces conformity, normalizes inequality, and perpetuates the belief that female efforts are natural, inevitable, and morally correct.

Participant 22 (married) sharing about herself said *“My mother always said we are women*,* we should not have much preferences or favorites… because at the end men run the house… learning to respect their wishes will make life easier. As a woman I feel those were the words of wisdom from her… girl and boys cannot be same… girl has to fulfil her household duty of kitchen*,* cleaning and raising children so she has to be taught all those and boys must be taught how to earn. Years after years people have been raised like that… what can we do with that. (irritated). We also do the same with our children.”*

This narrative reveals how socialization in context of females becomes internalized as ‘wisdom’ which is a survival strategy passed from mother to daughter under the guise of care and practicality. The participant accepts, rationalizes, and continues the same conditioning she received, validating the rigid division of labor between male and female. Her endorsement of these beliefs, despite the subtle irritation in her tone, indicates a cognitive dissonance—a psychological discomfort stemming from the clash between what she has been taught and what she may intuitively feel as unfair or restrictive.

Her statement *“We also do the same with our children”* shows how patriarchal norms are imitated not solely by men, but also by women who have been shaped by these ideologies themselves. This represents intergenerational transmission of gender inequality, where adaptation to oppression is passed on as tradition, making systemic issues seem natural and apolitical.

The narrative also explains how subservience and domestic confinement are framed as moral duties rather than imposed limitations. This further describes the position wherein, girls are groomed for obedience and boys are groomed for autonomy. It also entails how women learn to suppress personal desires (“we should not have much preferences or favourites”) and instead derive their value from fulfilling roles of service like wives, mothers, caretakers. Female disempowerment is sustained by the belief that “men run the house,” which is not challenged but rather acknowledged as a structural reality.

Moreover, the participant’s irritation suggests a subtle, perhaps unconscious, awareness of this injustice. It reflects the internal tension experienced when long-held beliefs start to collide with emerging self-awareness or shifting societal discourse. However, in the absence of supportive environments or critical consciousness, this discomfort is often silenced and buried, leading women to repeat the very practices that hurt them as there seems to be no other alternative.

This theme emphasizes that gender discrimination is frequently maintained through subtle, emotional, and commonplace practices that are taught in kitchens, whispered as advise, or perpetuated as tradition. It is not necessarily imposed brutally or explicitly. It encourages critical thinking about how internalized oppression can be facilitated by cultural continuity and how transformation requires questioning these ingrained standards. In addition to individual resistance, collective re-education, intergenerational communication, and the establishment of spaces where women can relearn, question, and rebuild their sense of identity outside of home responsibilities are all necessary to end this cycle.

### Theme 5: Male control over women's usurping females of their opportunities for education and work, and coercing into obedience

This theme explores how women, despite their competence and aspirations, are denied access to education and self-determined life choices due to patriarchal norms that prioritize obedience over autonomy. It emphasizes how internalized familial expectations serve as subtle but powerful forms of male control.

*Participant 9 (unmarried, age 19) said “ I and my brother have been raised differently*,* I have never ever seen him doing household chores*,* but I am always said by my parents that if I don’t learn to do all this what will my in-laws say in future that we did’t raise you properly. I cleared the entrance exam at my own merit but I was not allowed to go for counselling as the university is far from my place*,* while my brother was never denied…you tell in today’s changing world how would I feel…I feel so much frustrated….and now I am in a course I never wanted to opt…it feels so irritating…. It feels miserable actually…don’t these situations trigger girls who are really worthy but are stopped from doing so… it must be equally suffocating for them.*

This narrative exemplifies the pervasive nature of structural injustice for females, often disguised as familial concern. Despite securing admission to a prestigious institution through her own merit, the participant was denied the opportunity to attend counselling solely because the university was geographically distant, whereas, such restriction was never imposed on her brother. Such selective control underscores the male-female disparities embedded in the socialization process, wherein boys are granted autonomy while girls are subjected to constraint. Here the burden of compromise was disproportionately placed on women. Simultaneously, she was expected to acquire domestic skills in anticipation of her future role as a daughter-in-law, reinforcing the notion that a woman’s worth is measured by her conformity to traditional roles. Her description of feeling “frustrated,” “irritated,” “miserable,” and “suffocated” reveals the deep emotional toll of this systemic denial. This theme illuminates how gendered conditioning promotes compliance in girls and freedom in boys, resulting in enduring emotional distress and internal conflict among women who recognize the disparity between their potential and the limitations imposed upon them. These quiet injustices create chronic psychological distress that may perpetrate suicidal thoughts.

### Theme 6: Social isolation and lack of support in times of crisis

This theme explores the profound emotional suffering women experience within abusive, neglectful, or unsupportive domestic environments. This reflects the pain that is often silenced, dismissed, or normalized. In patriarchal rural settings, women’s distress frequently goes unacknowledged, with culturally entrenched expectations to “adjust” compounding their psychological vulnerability.

Participant 41 (widow, age 41) shared her harrowing journey following the death of her husband:

*“After my husband’s death, I was completely broken. I stayed for a few days in my in-laws’ house—it was the most difficult time. My child was just one year old… they blamed me for everything. For a widow, things become even more difficult. People started speaking less to me… I felt so lonely (sobs). I felt discriminated against too. In villages, they say many things about how widows should behave—these age-old restrictions. I thought about ending my life many times. I prayed to God to take me too. It was getting unbearable. Eventually, I came back to my mother’s place.”*

Her narrative reveals the social abandonment and emotional isolation widows often face. Rather than receiving empathy, she was subjected to blame, silence, and stigmatization. It reveals the culturally embedded view that a widow’s life becomes one of diminished value and increased restrictions. Her grief was not only personal but socially penalized, pushing her toward suicidal ideation.

Another similar instance, recounted by Participant 28 (married, age 29), describing her cousin whose life was overshadowed by chronic emotional neglect and domestic abuse:

*“Her in-laws always complained, no matter how much she did for them. They were never satisfied. Her husband was also an addict—he used to fight with her a lot. She told her mother many times that she was unhappy. But as usual, mothers say, ‘learn to adjust.’ They had a fight that night too… maybe she couldn’t take it anymore, and she hanged herself.”*

This account illustrates the cumulative trauma experienced by women trapped in cycles of emotional invalidation. Despite repeatedly seeking support, the woman’s pain was minimized by her family, echoing the normalized expectation to “adjust” instead of receiving support or intervention. The tragic outcome underscores how internalized cultural narratives particularly those that promote endurance over well-being can lead to fatal consequences.

The theme also reveals the subtle ways in which maternal complicity that has been shaped by generations of social conditioning and can perpetuate silence in females. Mothers, themselves products of similar oppression, may unintentionally reinforce harmful norms by discouraging their daughters from voicing discontent or seeking change.

Ultimately, this theme exposes the serious consequences of ignored mental health struggles, especially when women are denied the space to express, process, or seek help for their suffering. It calls for a critical re-examination of how emotional pain is culturally managed and highlights the urgent need for supportive, non-judgmental networks that prioritize women’s psychological well-being over social conformity.

## Discussion

The findings of this narrative inquiry shed critical light on the contextual, emotional, and cultural dimensions of suicidal vulnerability among rural Indian women. Drawing upon the rich, textured stories of 39 women across four villages in Bihar, the study reveals how suicidal thoughts, behaviours, and their meanings are narrated, interpreted, and culturally understood by rural women. These beliefs do not emerge in isolation; rather, they are deeply intertwined with women's life experiences, chronic invalidation, structural inequality, and cumulative emotional strain. This interpretation aligns with Suicide Scripts Theory [10], which conceptualizes suicide as a culturally embedded narrative shaped by socially shared beliefs about who is likely to engage in suicidal behaviour, the circumstances under which suicide becomes thinkable, and the meanings attributed to such acts. From this perspective, the present study does not document deaths by suicide themselves, but rather illuminates the culturally available scripts through which rural women interpret suffering, endurance, and suicide.

A striking theme that emerged across narratives was the normalization of women’s emotional pain. Emotional distress was frequently dismissed as an expected part of being a woman rather than recognized as a legitimate expression of suffering requiring acknowledgement or support. Participant 2’s statement, *“even if we cry… it hardly matters*,*”* reflects what Kleinman [27] has termed *social suffering*, wherein individual pain becomes embedded within broader social structures and cultural norms. Feminist theorists such as Bartky [28] and Bordo [29] have similarly argued that women are socialized into forms of *emotional docility*, whereby their emotional experiences are routinely minimized or disregarded. By normalizing the belief that “women cry” as an ordinary aspect of femininity, this cultural script delegitimizes women’s emotional experiences and effectively denies them the right to have their suffering acknowledged, validated, and responded to with care and support. Furthermore, such thinking reinforces gendered inequalities by silencing women’s voices and limiting their opportunities to seek help, thereby contributing to the broader social conditions that shape suicidal vulnerability.

Patriarchal socialization, reinforced through familial structures, significantly constrained the autonomy of women in the study. Participant 9’s narrative about being denied permission to attend university counselling, even after qualifying on her own merit while, on the other hand, her brother faced no such restriction. This illustrates the persistent male-female disparity in access to opportunities. This experience reflects broader patterns of educational deprivation and women's domestic confinement reported in prior scholarship [30, 31]. Mothers were sometimes positioned, often unknowingly, as transmitters of patriarchal ideology. Participant 22’s recollection of her mother’s advice and, urging acceptance of male-female responsibilities, demonstrates the feminist concept of intergenerational transmission of gender norms [32, 33]. It was observed that internalization of these norms resulted into a dual conflict of being both victims of oppression and agents of its continuation. This reflects what feminist scholars describe as a dilemma of complicity, where a sense of injustic may coexist with resignation of tradition [34].

The denial of autonomy was not limited to education alone. Narratives revealed how early marriage, overwhelming domestic responsibilities, and cultural expectations pushed many women into profound distress by restricting their ability to make decisions about their own lives. Participant 36, for instance, described how her academic aspirations were curtailed by forced marriage and caregiving roles. Such structural constraints echo Young’s articulation of gendered oppression [35], wherein women are not only denied choices but are stripped of the imagination of alternative futures. Feminist analyses of structural inequality further highlight how domestic responsibilities, although framed as familial obligations, can become mechanisms of social control when they systematically limit women’s educational, economic, and personal development [36, 37]. These narratives illustrate how violations of women’s rights, such as right to education, autonomy, self-determination, and equal opportunities, are a part of everyday family and community practices in rural settings. Rather than reflecting individual vulnerability alone, such violations create structural conditions that may intensify women’s vulnerability to suicide-related distress. Consistent with the findings of Cai et al. [38], the participants’ accounts demonstrate that gender-discriminatory laws, policies, and sociocultural structures are lived realities that shape women’s everyday experiences of distress. The denial of autonomy, normalization of suffering, and emotional suppression described by participants further illustrate how structural inequalities contribute to suicidal vulnerability. Similar understandings of suicide as an act of protest, communication, or moral expression have also been documented among women in rural China [21] and Sri Lanka [18, 20], suggesting that these narratives reflect broader gendered suicide scripts across low- and middle-income country contexts.

The psychological consequences of sustained emotional suppression were articulated poignantly through metaphors like the “overflowing pot,” used by Participant 4. This metaphor captures the silent, slow accumulation of unaddressed distress that mayreach a crisis point. Riessman [25] highlights how metaphors in narratives provide access to emotional thresholds and unspoken suffering. The internalization of shame, especially in relation to infertility or emotional neglect, reflects what Brown [39] termed “insidious trauma”. Such trauma has been described as arising from the chronic, everyday mortifications of discriminated existence. It has been asserted that emotional labour, when unrecognized and unsupported, becomes a heavy psychic burden [40] that may help explain why participants narrated suicide-related distress through the language of escape, protest, and constrained agency. The internalization of shame observed in these narratives resonates with findings from Sri Lanka, where suicide scripts among young women are deeply intertwined with embodied shame and gendered expectations [18].

Widowhood emerged as an important context of vulnerability to suicide-related distress among rural women. Participant 41’s narrative illustrates how widowhood extends beyond personal bereavement to become a socially imposed condition marked by blame, discrimination, and exclusion. These narratives suggest that widows are often denied fundamental rights to dignity, equality, social participation, and freedom from discrimination through practices that normalize blame, stigma, and exclusion. Although Durkheim’s theory of egoistic suicide proposes that weakened social integration increases suicidal vulnerability [41], the present findings suggest that vulnerability extends beyond the absence of social ties. Instead, participants described social relationships that actively reinforced gendered discrimination and curtailed women’s rights and agency. Rather than serving as a source of protection, family and community structures often perpetuated surveillance, exclusion, and unequal power relations, creating social conditions that intensified suffering and heightened vulnerability to suicidality.

Another distressing dimension involved the culture of “adjustment,” where women were instructed to endure emotional abuse or domestic violence without complaint. Participant 28 described how her relative, trapped in a marriage with an abusive addict, was advised repeatedly to “adjust” until she eventually died by suicide. This normalization of endurance is consistent with Mohanty’s postcolonial feminist critiques that argue that systemic violence is frequently concealed by culturally acceptable resilience [42], which demonises help-seeking and sanctifies silence.

Themes of surveillance and gendered behavioral regulation recurred across narratives. Participant 18’s account of societal scrutiny over girls' speech, mobility, or everyday behavior reflects Foucault’s notion of disciplinary power [43]. Women and girls are disproportionately targeted by this type of surveillance, which restricts their freedom and shapes their subjectivity, as feminist researchers like Fraser [44] and Harding [45] have demonstrated. Here, Bartky’s idea of “internalized oppression” is essential: women start to modify their own behaviors, aspirations, and goals to conform to societal expectations [28]. Recent qualitative work in Nepal further demonstrates that suicide scripts are gender-differentiated, with women’s narratives emphasizing relational suffering, constraint, and moral positioning, closely aligning with the patterns observed in the present study [19].

These narratives frame suicide not merely an expression of psychological disorder, but as a socio-cultural phenomenon. Specifically, it is constructed as a response to prolonged systemic disenfranchisement. As Jackson [46] contends, narratives function not only as reflections of individual experience but also as means of contesting dominant ideologies. Viewed through this lens, the participants’ accounts of suicidal ideation and emotional suffering emerge as critical commentaries on a social order that routinely marginalizes their pain. Within these narratives, suicide emerges as a culturally intelligible script through which distress, constrained agency, and gendered oppression are simultaneously communicated. Feminist scholars argue that such narratives should not be dismissed as indicators of individual weakness, but rather recognized as powerful, albeit deeply painful, testimonies to the cumulative psychological toll of navigating oppressive socio-cultural structures [47, 48].

### Implications, limitations and policy recommendations

The implications of these findings are profound for both mental health practice and public policy. These findings suggest that suicide is constructed as a culturally scripted form of escape from and protest against lives marked by usurped agency. There is a pressing need to reconceptualize mental health care in rural India through cultural script lens. Such a viewpoint recognizes the socio-political dimensions of psychological distress. Mental health interventions must move beyond individual-focused approaches to engage with the collective, cultural, and structural factors that shape women’s lived experiences. These conditions reflect not only psychosocial distress but also systematic violations of women’s human rights, including the denial of autonomy, mobility, and dignity. Hence, programs must empower women by offering safe spaces for emotional expression, community support, and narrative agency. Efforts must also focus on engaging mothers and elder women as key stakeholders in the process of dismantling internalized patriarchy. Establishing local language-based, anonymous mental health helplines that specifically address women’s issues, such as marital abuse, infertility, or social isolation may help vulnerable individuals.

Additionally, policies aimed at preventing early marriage, promoting girls’ education, and supporting widows and survivors of domestic violence must be implemented rigorously. Public health messaging must confront the cultural script of endurance, which normalizes suffering and silences resistance. There is a pressing need for village-level enforcement of the Prohibition of Child Marriage Act (2006) and for programs that sensitize parents about the psychological harm caused by early marriage, denied mobility, and career suppression. 

Gender-sensitization efforts must not only target men but also empower women to question inherited roles. Mental health literacy campaigns should be designed in culturally resonant ways, encouraging community members to recognize distress not as personal weakness but as a call for support and change. There is a need to develop and implement decentralized suicide prevention programs, involving frontline workers like ASHAs, ANMs, and Anganwadi workers. These workers can be trained to identify early signs of emotional crisis and refer women to accessible help.

However, the study is not without its limitations. The regional specificity of the research focused on four villages in Bihar, limits the generalizability of findings to other rural or urban areas in India. Further, self-selection bias may have influenced the sample, as women more willing to articulate emotional pain may have been more likely to participate. Group settings, while useful for shared narratives, may have restricted the expression of deeply personal or traumatic experiences due to the presence of others. Some narratives were fragmented or emotionally interrupted, which Riessman [25] identifies as a common challenge in co-constructed storytelling. Future studies can integrate Focus Group Discussions with in-depth interviews to balance the exploration of collective cultural meanings with more nuanced, individual-level narratives. Furthermore, the absence of male perspectives in the dataset limits our understanding of how these separate female-male norms are reinforced or challenged by male family members and community actors. Finally, the study’s lack of follow-up or long-term psychological support for participants highlights the ethical complexity of narrative research in emotionally sensitive domains.

Future research must address these limitations by incorporating a broader demographic sample across multiple regions and including intergenerational and gender-inclusive dialogues. Longitudinal studies tracking how girls internalize or resist gender scripts over time would add important depth. Further exploration into resilience narratives, including, how some women manage to survive and thrive despite similar constraints. This would help in designing strength-based interventions. Investigating the role of community-based peer support, digital platforms, or informal care networks could illuminate pathways for sustainable mental health care in rural contexts.

## Conclusion

This study offers a deeply humanizing and contextually grounded perspective on suicidal distress among rural Indian women. By centering women’s narratives, it becomes evident that suicide-related distress should not be understood solely as the product of isolated despair. Rather, participant's narratives framed suicide among women as a culturally scripted response to patriarchal systems. This study’s findings, together with related evidence, challenge us to rethink suicidality among women through the lenses of human rights and social justice. Addressing women’s suicidal vulnerability therefore requires more than mental health care; it also demands structural transformation and recognition of suicide-related distress as a response to gendered injustice and human-rights violations.

## Supplementary Information

Supplementary Material 1.

## Data Availability

Due to the sensitive nature of the data and to ensure participant confidentiality, the dataset used in this study is not available for public access.
