# Supplementary material for: Indian rural women’s suicide beliefs and experiences: female suicidality as escape from and protest against a life usurped of agency
Source: BMC Womens Health. 2026 Sep 14;26:466. doi: 10.1186/s12905-026-04676-9 (PMC13591895; doi:10.1186/s12905-026-04676-9)
Supplement: Supplementary file 1 [file 12905_2026_4676_MOESM1_ESM.docx]

*Supplementary Material*

Ghosh D, Verma SK, ALmerab MM, Mamun MA, Mandal SP, Bhattacharya S, Raj S. Indian rural women’s suicide beliefs and experiences: female suicidality as escape from and protest against a life usurped of agency. BMC Women’s Health. 2026. <https://doi.org/10.1186/s12905-026-04676-9>

**Supplementary Table S1. Consolidated Criteria for Reporting Qualitative Research (COREQ) checklist**

| **No.** | **Item** | **Guide Questions/Description** | **Reported** |
| --- | --- | --- | --- |
| **Domain 1: Research Team and Reflexivity** | | | |
| **Personal Characteristics** | | | |
| 1 | Interviewer/facilitator | Which author/s conducted the interview or focus group? | Methods – D.G., S.B., and S.R. conducted fieldwork and facilitated the focus group discussions (FGDs). |
| 2 | Credentials | What were the researcher’s credentials? | Methods – S.R. (PhD), S.B. (PhD scholar), D.G. (PhD), S.K.V. (PhD), and M.A.M. (MRes). |
| 3 | Occupation | What was their occupation at the time of the study? | Methods – Researchers were faculty members or doctoral researchers in social sciences/public health/psychology. |
| 4 | Gender | Was the researcher male or female? | Methods/Reflexivity – FGD facilitation was led by female researchers/facilitators (D.G., with support from S.B.). |
| 5 | Experience and training | What experience or training did the researcher have? | Methods – Researchers had training in qualitative research; facilitators were familiar with FGD ethics and trauma-informed interviewing. One team member was a licensed clinical psychologist. |
| **Relationship with Participants** | | | |
| 6 | Relationship established | Was a relationship established prior to study commencement? | Methods – Participants were approached through local community gatekeepers; no prior relationship with the research team was reported. |
| 7 | Participant knowledge of the interviewer | What did the participants know about the researcher? | Methods – Participants were informed about the purpose, goals, and use of the research in local language. |
| 8 | Interviewer characteristics | What characteristics were reported about the interviewer/facilitator? | Reflexivity – Female researchers/facilitators emphasised empathy, local cultural familiarity, and non-hierarchical facilitation. |
| **Domain 2: Study Design** | | | |
| **Theoretical Framework** | | | |
| 9 | Methodological orientation and theory | What methodological orientation was stated? | Methods – Narrative inquiry using a feminist and sociocultural lens. |
| **Participant Selection** | | | |
| 10 | Sampling | How were participants selected? | Methods – Purposive sampling |
| 11 | Method of approach | How were participants approached? | Methods – Through the village sarpanch and anganwadi workers. |
| 12 | Sample size | How many participants were in the study? | Methods – Thirty-nine women participated in four FGDs, with 7–11 participants in each group. |
| 13 | Non-participation | How many people refused or dropped out? | Methods – Forty-two women were approached and 39 consented; therefore, three did not participate. |
| **Setting** | | | |
| 14 | Setting of data collection | Where was the data collected? | Methods – FGDs were conducted in safe, locally accessible community settings in the four selected villages in Bihar; privacy and comfort were prioritised. |
| 15 | Presence of non-participants | Was anyone else present? | Methods – Only participants and researchers/facilitators were present during the FGDs. |
| 16 | Description of sample | What are the important characteristics of the sample? | Methods – Rural adult women aged 18–47 years from four villages in Bihar; participants included 21 unmarried women, 16 married women, and 2 widows. |
| **Data Collection** | | | |
| 17 | Interview guide | Were questions, prompts, guides provided? Was it pilot tested? | Methods – A semi-structured FGD guide with open-ended, culturally sensitive questions was used; see Supplementary Table S2. |
| 18 | Repeat interviews | Were repeat interviews carried out? | NA – No repeat FGDs were conducted; each participant took part once. |
| 19 | Audio/visual recording | Did the research use recording? | Methods – FGDs were audio-recorded with consent; participants provided verbal or written informed consent, as appropriate. |
| 20 | Field notes | Were field notes made? | Methods – Detailed field notes captured nonverbal cues, group dynamics, and emotional responses. |
| 21 | Duration | What was the duration of the interviews or FGDs? | Methods – FGDs lasted approximately 90 minutes. |
| 22 | Data saturation | Was data saturation discussed? | Not explicitly reported in the main manuscript. |
| 23 | Transcripts returned | Were transcripts returned to participants? | NA – Due to literacy and logistical issues, transcripts were not returned. |
| **Domain 3: Analysis and Findings** | | | |
| **Data Analysis** | | | |
| 24 | Number of data coders | How many coded the data? | Methods – Initial coding by D.G. and S.R.; themes were reviewed collaboratively by the wider author team. |
| 25 | Description of coding tree | Was a coding tree provided? | NA – Themes described narratively; no hierarchical tree included. |
| 26 | Derivation of themes | Were themes identified in advance or derived from the data? | Methods – Emergent themes through inductive, narrative-based analysis. |
| 27 | Software | What software was used? | NA – Manual coding with spreadsheets and theme mapping. |
| 28 | Participant checking | Did participants provide feedback on findings? | NA – Findings were not returned to participants because of privacy, logistical, and emotional safety considerations. |
| **Reporting** | | | |
| 29 | Quotations presented | Were participant quotations used? | Results – Yes, verbatim quotations from FGD participants were included. |
| 30 | Data and findings consistent | Were data and findings consistent? | Results – Yes, quotes strongly support each theme and subtheme. |
| 31 | Clarity of major themes | Were major themes clearly presented? | Results – Yes; six major themes were presented: (1) normalization of women’s suffering within family and community life; (2) enduring chronic distress in silence under expectations; (3) restrictions on women’s autonomy and life choices; (4) intergenerational reproduction of patriarchal gender norms; (5) male control over women’s opportunities for education and work and coercion into obedience; and (6) social isolation and lack of support in times of crisis. |
| 32 | Clarity of minor themes | Were minor/divergent themes discussed? | Results – Yes; narratives included minority or divergent elements such as resistance, questioning inherited gender norms, community/family support, and coping possibilities. |

**Supplementary Table S2. Focus Group Discussion Guide: Core Questions**

| **Core domain** | **Questions** |
| --- | --- |
| Daily life and responsibilities | “Can you share what a typical day in your life looks like?” / “What roles and responsibilities do women usually have in your community?” |
| Emotional distress | “What kinds of problems or pressures do women in your community usually face?” |
| Gendered expectations | “What is expected from women in terms of behaviour, responsibility, and tolerance?” |
| Expression of distress | “How do women usually express emotional pain or distress?” |
| Silence and support | “Do women talk about their problems openly? If not, why?” / “Who do women usually turn to when they are deeply hurt or troubled?” |
| Beliefs about suicide | “In your opinion, what factors may contribute to suicide among women?” |
| Community response | “How do people in the community respond when suicide occurs?” |
| Meanings of suicide | “What meanings do women or community members attach to suicide in a woman’s life?” |
| Coping alternatives | “What options or forms of support are available when women feel overwhelmed or distressed?” |
